# Supplementary material for: Rates and reasons for hospital readmission after acute ischemic stroke in a US population-based cohort
Source: PLoS One. 2023 Aug 3;18(8):e0289640. doi: 10.1371/journal.pone.0289640 (PMC10399731; doi:10.1371/journal.pone.0289640)
Supplement: S1 Fig — (PDF) [file pone.0289640.s001.pdf]

S1 Fig Example diagnostic plots showing violations of assumption of proportional hazards for hypertension with top panel showing non-parallel lines on log-log plot of survival and bottom panel showing increasing scaled Schoenfeld residuals over time (more prominent in early period)

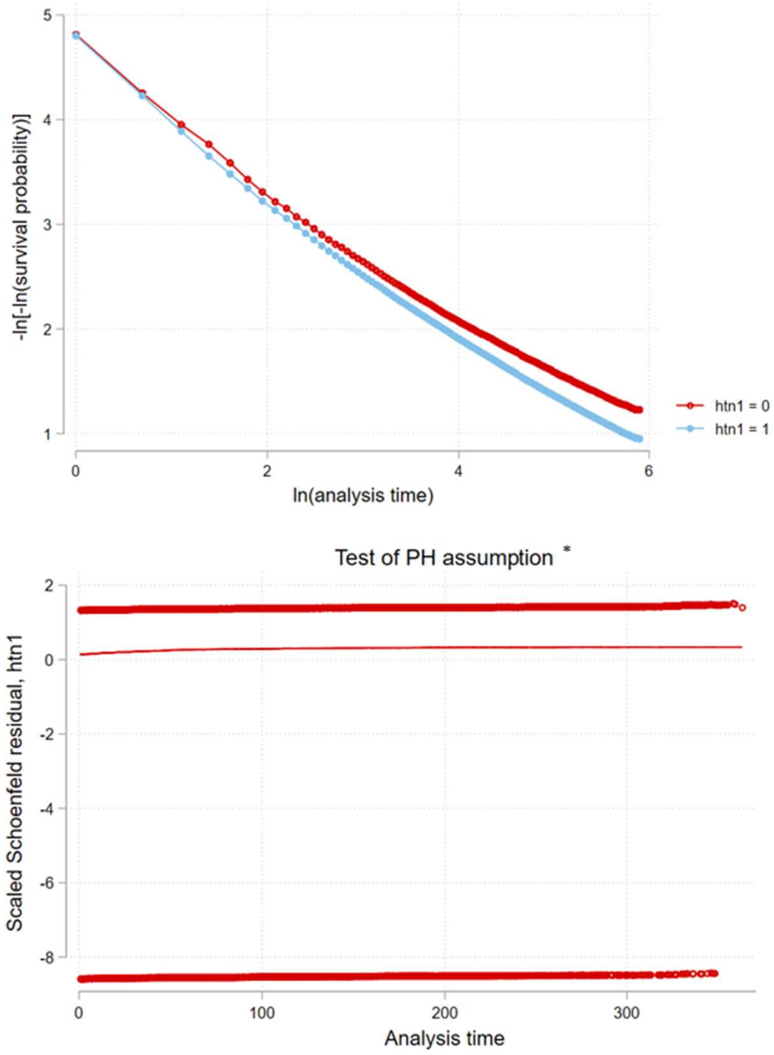

*Proportional hazards global test Chi-square(1) 42.38, p>0.001
