# Supplementary material for: Rates and reasons for hospital readmission after acute ischemic stroke in a US population-based cohort
Source: PLoS One. 2023 Aug 3;18(8):e0289640. doi: 10.1371/journal.pone.0289640 (PMC10399731; doi:10.1371/journal.pone.0289640)
Supplement: S1 Table — (PDF) [file pone.0289640.s002.pdf]

S1 Table. Principle Diagnosis at Readmission after Discharge with Ischemic Stroke by ICD Category

ICD Code Starting with	Disease Category	Frequency	Percentage
I	Circulatory System	6,516	34.50%
A/B	Infectious and Parasitic Diseases	1,943	10.30%
S/T	Injury, Poisoning, Certain Other Consequences of External Causes" 19 "No codes listed, will be used for emergency code additions	1,746	9.30%
K	Digestive System	1,612	8.50%
N	Genitourinary System	1,303	6.90%
J	Respiratory System	1,221	6.50%
G	Nervous System	1,077	5.70%
R	Symptoms, Signs and Abnormal Clinical and Lab Findings	952	5.00%
E	Endocrine, Nutritional, Metabolic	850	4.50%
F	Mental and Behavioral Disorders	380	2.00%
M	Musculoskeletal and Connective Tissue	362	1.90%
D	Neoplasms, Blood, Blood-forming Organs	319	1.70%
L	Skin and Subcutaneous Tissue	246	1.30%
C	Neoplasms	233	1.20%
H	Eye and Adnexa, Ear and Mastoid Process	52	0.30%
V/W/X/Y	External Causes of Morbidity" 21 "Factors Influencing Health Status and Contact with Health Services	29	0.20%
Q	Congenital Malformations, Deformations and Chromosomal Abnormalities	16	0.10%
O	Pregnancy, Childbirth and the Puerperium	4	0.00%
	Missing	5	0.00%
	Total	18,866	
